# Using Revolution™ CT Angiography to Assess Complex Coarctation of the Aorta in Infants and Its Association with a Prolonged Postoperative Cardiac ICU Stay

**DOI:** 10.21470/1678-9741-2022-0402

**Published:** 2023-07-14

**Authors:** Hui-Jun Xiao, A-Lai Zhan, Rui-Gang Huang, Wei-Hua Lin, Qing-Wen Huang

**Affiliations:** 1 Department of Radiology, Zhangzhou Affiliated Hospital of Fujian Medical University, Zhangzhou, People’s Republic of China

**Keywords:** Artificial Respiration, Aortic Coarctation, Angiography, Reproducibility of Results, Risk Factors

## Abstract

**Objective:**

To investigate the accuracy of aortic dimensions measured by Revolution™ computed tomography (CT) in infants with complex coarctation of the aorta (CoA) and to further analyze the utility of the degree of CoA in predicting the risk of prolonged postoperative cardiac intensive care unit stay.

**Methods:**

A total of 30 infants with complex CoA who underwent surgical correction from January 2020 to July 2022 were retrospectively enrolled. General demographic data, preoperative imaging, and perioperative outcomes were collected. Univariate and multivariate analyses were performed to investigate predictors of prolonged postoperative cardiac intensive care unit stay, and the reliability of the CT measurements was assessed by the intraclass correlation coefficient.

**Results:**

All infants were divided into a mild or severe CoA group. The duration of mechanical ventilation and cardiac intensive care unit stay in the mild CoA group were significantly lower than those in the severe CoA group. After multivariate analysis, we found that the degree of CoA and age at surgery were significant predictors of prolonged postoperative cardiac intensive care unit stay. The intraclass correlation coefficient between CT measurements and intraoperative measurements was between 0.937 and 0.975, and the measurement results had good reliability.

**Conclusion:**

CT angiography can provide a comprehensive and accurate preoperative evaluation of aortic dimensions measured in infants with complex CoA. The degree of CoA is an independent risk factor for prolonged postoperative cardiac intensive care unit stay in infants with complex CoA.

## INTRODUCTION

**Table:** 

Abbreviations, Acronyms & Symbols				
ARF	= Anterior right foot position		DSCT	= Dual-source computed tomography
AS	= Aortic stenosis		FPL	= Foot posterior left position
ASD	= Atrial septal defect		HAR	= Head anterior right position
CDR	= Coarctation site-diaphragm ratio		ICC	= Intraclass correlation coefficient
CI	= Confidence interval		PDA	= Patent ductus arteriosus
CICU	= Cardiac intensive care unit		PLH	= Posterior left head position
CoA	= Coarctation of the aorta		SD	= Standard deviation
CT	= Computed tomography		TTE	= Transthoracic echocardiography
CTA	= Computed tomography angiography		VSD	= Ventricular septal defect
DORV	= Double outlet right ventricular			

Coarctation of the aorta (CoA) is a common type of congenital heart disease, in which the main lesion is coarctation of the aortic isthmus near the ductus arteriosus. CoA can manifest as localized coarctation or a long segment of arcuate aortic hypoplasia, which can also be accompanied by other intracardiac or extracardiac malformations, such as aortic arch hypoplasia, ventricular septal defect (VSD), and patent ductus arteriosus (PDA)^[1-3]^. With the significant improvement of surgical techniques, anesthesia concepts, and cardiopulmonary bypass technology in recent years, the use of median thoracotomy to correct complex CoA has achieved good short-term and long-term results, even in neonates with low age, low weight, or preterm infants^[4,5]^. The operative mortality rate of CoA has been reduced to 1%, according to previous large cohort studies^[6]^. Therefore, identifying and understanding the factors associated with prolonged cardiac intensive care unit (CICU) stay after CoA surgery have become a new focus in recent years because modifying these factors can further improve a patient’s prognosis and reduce medical resource utilization. A study by Phillip Burch et al.^[7]^ showed that low birth weight was not a factor for adverse events after aortic surgery in neonates or infants younger than three months. Ugo Giordano et al.^[8]^ noted that the incidence of hypertension after complex coarctation surgery was higher than that after simple coarctation. However, there are few relevant studies on the influence of the degree of coarctation of infants with complex CoA on the length of hospital stay after surgical correction. Therefore, we conducted a retrospective analysis of the effect of the degree of coarctation on postoperative CICU stay in infants with complex CoA and further analyzed the accuracy of Revolution™ CT angiography in measuring aortic dimensions in infants with complex CoA.

## METHODS

We retrospectively identified the records of 30 infants who underwent surgical correction for complex CoA from January 2020 to June 2022. These infants underwent computed tomography angiography (CTA) by Revolution™ computed tomography (CT) before surgery. Complex CoA was defined as CoA with other intracardiac or extracardiac malformations, such as atrial septal defect, VSD, and PDA. Using the results of CTA, the ratio of the size of the aortic isthmus to the size of the descending aorta at the level of the diaphragm (CDR) was used to describe the degree of aortic arch coarctation, where 50% < CDR < 75% was defined as mild coarctation and CDR ≤ 50% was defined as severe coarctation^[9]^. A total of 16 infants with mild coarctation and 14 with severe coarctation were included in this study cohort. Because this study was retrospective, the institutional ethics review board approved the request for a waiver of written informed consent for this study (approval number 2022LWB270). Inclusion criteria were as follows: 1) the infants were diagnosed with complex CoA and received surgical correction; 2) all patients were less than one year old at the time of surgery; and 3) all infants received CTA by Revolution™ CT before the operation. Exclusion criteria were as follows: 1) cases with insufficient clinical data required for the study; 2) cases diagnosed with simple CoA; and 3) cases combined with congenital diseases of other systems except for cardiovascular disease. Surgical correction decision criteria were individualized according to the condition of each infant including: intracardiac or extracardiac malformations, with left ventricular hypertrophy and hypertension, luminal diameter of the coarctation < 50% of the aortic diameter at the level of the diaphragm, and peak transient gradient at the coarctation > 20 mmHg^[10,11]^.

### Scanning Protocol

**Fig. 1:**
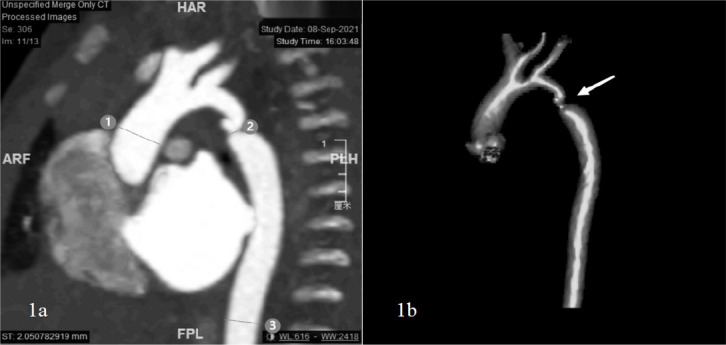
Computed tomography images of a 10-day-old boy with coarctation of the aorta. A) Ascending aorta (1); aortic isthmus (2); descending aorta at level of diaphragm (3). B) View of the aortic isthmus after three-dimensional reconstruction (arrow). ARF=anterior right foot position; FPL=foot posterior left position; HAR=head anterior right position; PLH=posterior left head position.

The infants in this study underwent CTA with a 256-row Revolution™ CT from General Electric Company before surgery. Before the examination, the subjects’ parents signed the informed consent form for the CTA. All infants needed to indwell an intravenous catheter in the upper or lower extremities and be sedated by oral administration of an appropriate amount of 10% chloral hydrate (0.5 mL/kg) approximately 20 minutes before the examination. A lead apron was used to cover and protect the patients’ pelvic area, which did not need to be examined. The scanning parameters in this study were set as follows: tube voltage of 70-80 kV, collimator width 40 mm, gantry rotation time of 0.28-0.30 s, and pitch of 0.531-0.984. Scans were performed in a craniocaudal direction, from the thoracic entrance to 2 cm below the level of the diaphragm. Angiographic contrast agent (iopromide, 100 ml: 62.34 g, Bayer, Germany) was injected into all infants at a rate of 0.8-1.5 mL/s through the upper or lower extremity vein. The reconstructed images were based on a slice thickness of 0.625 mm and increments of 0.625mm. The scanned image data were analyzed on a General Electric AW 4.7 processing workstation. Two experienced radiologists analyzed the reconstructed images. The widths of three levels, including the ascending aorta, the aortic isthmus, and the descending aorta at the diaphragm level, were measured in the reconstructed images. If the two analyses diverged, the two radiologists discussed their results and agreed (Figure 1).

### Surgical Methods

After median sternotomy was performed and cardiopulmonary bypass was successfully established, the ascending aortic arch and branch vessels were carefully identified and separated, the location of coarctation was dissected, and the diameter of the ascending aortic arch and aortic isthmus was measured with small calipers under direct vision. In the discrete type, simple continuous end-to-end anastomosis was performed after complete resection of all coarctation and ductal tissue. If combined with a hypoplastic aortic arch, coarctation and ductal tissue resection plus end-to-end extension anastomosis and the patch expansion technique were used if necessary. The patient was transferred to the CICU after the operation.

### Data Collection

Sex, gestational age, associated cardiovascular complications, age at surgery, weight at surgery, and other preoperative clinical data of all infants were collected from the database. Intraoperative data, such as aortic cross-clamping time, cardiopulmonary bypass time, and surgical duration, were collected. The ascending aorta and aortic isthmus dimensions in the operation were collected by retrieving surgical records as the gold standard. Postoperative data such as postoperative pneumonia, duration of mechanical ventilation, and length of CICU stay were also collected. The patients’ sensitive information was kept entirely confidential in this study, and all information was used for this study only. All information irrelevant to this study, such as the patients’ names, has been deleted.

### Statistical Analysis

Qualitative variables are expressed as frequencies and percentage values (%), and quantitative variables are defined as the mean±standard deviation. The chi-square test or Fisher’s precision probability test was used to analyze categorical variables. If the quantitative variables conformed to the normal distribution after the normal test, the independent sample *t*-test was used. If not, a nonparametric test was used for analysis. Predictors associated with prolonged CICU stay after complex CoA surgery were assessed by univariate and multivariate analysis models. The interobserver reliability of Revolution™ CT measurements was evaluated by intraclass correlation coefficients (ICCs) and stratified according to the findings of Koo and Li (< 0.5, poor reliability; 0.50-0.75, reliability; 0.75-0.90, good reliability; 0.90-1, excellent reliability)^[12]^. ICC and Bland‒Altman plots were used to examine the agreement between Revolution™ CT and intraoperative measurements. All statistical analyses were performed using IBM Corp. Released 2013, IBM SPSS Statistics for Windows, version 22.0, Armonk, NY: IBM Corp. and GraphPad Prism (version 8.0, GraphPad Software, California, United States of America). When the *P*-value was < 0.05, it was considered statistically significant.

## RESULTS

A total of 30 infants with complex CoA were included in this study - 16 infants with mild coarctation and 14 infants with severe coarctation. The ages of the two groups were 2.1±0.9 months and 2.4±1.2 months, respectively. VSD (90.0%) and PDA (40.0%) were the most common cardiovascular abnormalities in these infants. After statistical analysis, there was no statistically significant difference between the two groups in the general preoperative condition and relevant data during the operation (Table 1).

**Table 1:** Comparison of preoperative general clinical data between the two groups.

	Mild CoA group (n=16)	Severe CoA group (n=14)	*P*-value
Gender, n (%)			
Male	10 (62.5)	8 (57.1)	0.765
Female	6 (37.5)	6 (42.9)	
Gestational age, weeks	36.9±1.9	37.4±2.3	0.523
Associated cardiovascular complications, n (%)			
PDA	7 (43.8)	5 (35.7)	0.910
VSD	14 (87.5)	13 (57.1)	
ASD	5 (31.3)	4 (28.6)	
AS	2 (12.5)	1 (7.1)	
DORV	0 (0)	1 (7.1)	
Age at surgery, months	2.1±0.9	2.4±1.2	0.468
Weight at surgery, kg	4.3±0.9	4.4±1.1	0.944
Preoperative mechanical ventilation, n (%)	2 (10.5)	4 (18.2)	0.378
Surgical technique, n (%)			
Simple end-to-end anastomosis	16 (100)	12 (85.7)	0.226
End-to-end extension anastomosis/patch expansion	0 (0)	2 (14.3)	
Aortic cross-clamping time, min	97.7±18.5	95.4±19.4	0.740
Cardiopulmonary bypass time	134.9±20.2	131.4±21.2	0.647
Surgical duration, hours	4.2±0.9	4.2±0.8	0.882
Delayed sternal closure	1 (6.3)	2 (14.3)	0.586

AS=aortic stenosis; ASD=atrial septal defect; CoA=coarctation of the aorta; DORV=double outlet right ventricular; PDA=patent ductus arteriosus; VSD=ventricular septal defect

After analyzing the postoperative data in Table 2, it was found that there was no statistically significant difference in the incidence rates of postoperative pneumonia, feeding intolerance, chylothorax, and wound complications between the two groups. The duration of mechanical ventilation and the length of CICU stay in the mild CoA group were significantly lower than those in the severe CoA group. In addition, the incidence of recoarctation after surgical correction in the mild CoA group was not significantly different from that in the severe CoA group.

**Table 2:** Comparison of postoperative clinical results between the two groups.

	Mild CoA group (n=16)	Severe CoA group (n=14)	*P*-value
Postoperative pneumonia, n (%)	6 (37.5)	7 (50.0)	0.713
Feeding intolerance, n (%)	3 (18.8)	3 (21.4)	1.000
Chylothorax, n (%)	0 (0)	1 (7.1)	0.467
Wound complication, n (%)	3 (18.8)	2 (14.3)	1.000
Duration of mechanical ventilation, days	3.9±0.8	5.2±0.9	0.002
Length of CICU stay, days	6.1±1.4	8.4±1.8	0.001
Mortality, n (%)	0 (6.3)	1 (14.3)	0.467

CICU=cardiac intensive care unit; CoA=coarctation of the aorta

**Fig. 2:**
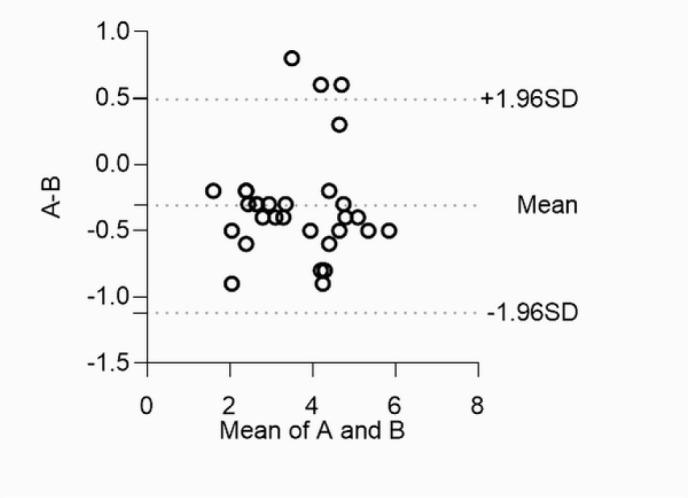
Bland-Altman plot of aortic isthmus dimensions measured by Revolution™ computed tomography vs. intraoperative measurements. A) Revolution™ CT measurements; B) intraoperative measurements. SD=standard deviation.

Table 3 shows the results from the univariate analysis. Prolonged postoperative CICU stay was closely associated with the degree of coarctation and duration of mechanical ventilation. After multivariate analysis, it was found that age at surgery, degree of CoA, and duration of mechanical ventilation were significant predictors of prolonged postoperative CICU stay. The ICC suggested that the results of Revolution™ CT and intraoperative measurements were good and consistent (ICC: 0.937-0.975), and the data were statistically significant (Table 4). ICC results showed that the Revolution™ CT measurement was more accurate than the echocardiographic measurement. Bland‒Altman plot analysis showed that the 95% limits of localization difference (Revolution™ CT measurements minus intraoperative measurement) between Revolution™ CT measurement and intraoperative measurement were -1.12 to 0.49 mm (Figure 2).

**Table 3:** Results of univariate and multiple analyses for length of CICU stay.

Predictors	Univariate		Multivariate	
	Beta (95% CI)	*P*-value	Beta (95% CI)	*P*-value
Gender	-0.04(-1.69, 1.38)	0.837	0.01 (-1.07, 1.11)	0.966
Age at surgery	-0.32 (-1.30, 0.10)	0.089	-0.39 (-1.42, -0.06)	0.035
Weight at surgery	-0.22 (-1.24, 0.32)	0.227	0.07 (-0.56, 0.86)	0.668
Severity of CoA	0.59 (1.11, 3.57)	0.001	0.40 (0.18, 2.99)	0.028
Duration of mechanical ventilation	0.67 (0.70, 1.78)	0.000	0.39 (0.07, 1.39)	0.033

CI=confidence interval; CICU=cardiac intensive care unit; CoA=coarctation of the aorta

**Table 4:** Concordance between Revolution™ CT measurements and intraoperative measurements.

Comparison group	ICC	*P*-value
Exam 1^*^ *vs.* intraoperative measurements	0.975	0.000
Exam 2^*^ *vs.* intraoperative measurements	0.937	0.000
Exam 3^*^ *vs.* intraoperative measurements	0.912	0.000
Exam 4^*^ *vs.* intraoperative measurements	0.873	0.000

CT=computed tomography; ICC=intraclass correlation coefficient ^*^Exam 1: dimensions of ascending aorta as measured by CT; Exam 2: dimensions of aortic isthmus as measured by CT; Exam 3: dimensions of ascending aorta as measured by echocardiography; Exam 4: dimensions of aortic isthmus as measured by echocardiography

## DISCUSSION

To our knowledge, this is the first study to examine the accuracy of Revolution™ CT in measuring aortic dimensions in infants with complex CoA and to further analyze the utility of CoA degree in predicting the risk of prolonged postoperative CICU stay. Additionally, previous studies on the postoperative prognosis of CoA included an extensive age range of participants, whereas our study only included infants^[13]^. This study allowed us to assess the risk of prolonged postoperative CICU stay in infants with complex CoA. Our results showed that the duration of mechanical ventilation and length of CICU stay in the mild CoA group were significantly lower than those in the severe CoA group, and the degree of CoA was an important predictor of prolonged postoperative CICU stay. In addition, this study also showed that Revolution™ CT had good reliability in measuring aortic dimensions.

This study identified the anatomical structural factors contributing to poor postoperative outcomes in infants with complex CoA. The degree of coarctation was an independent risk factor for prolonged postoperative CICU stay, which might be related to changes in collateral circulation function and hemodynamic pressure^[14,15]^. Although most of these anatomical structural risk factors were not subject to prior intervention, surgeons could expect prolonged CICU stay in infants with complex CoA with these risk factors by knowing this information in advance. This result could be further used as a sign of increased disease burden, to a certain extent, to provide the parents of infants with psychological preparation for surgery. In addition, we found that age at surgery was another independent risk factor for prolonged CICU stay in the infants in this cohort. This finding was consistent with previous research findings^[16]^. Previous studies on the surgical outcome of CoA have shown that preterm birth and concomitant systemic genetic abnormalities are independent risk factors for prolonged postoperative duration^[17]^. The study by Rinske IJsselhof et al.^[18]^ noted that simple CoA in the neonatal period would lead to a prolonged postoperative hospital stay. Our results also suggested that this phenomenon applies to infants with complex CoA.

Several imaging modalities help surgeons understand intracardiac and extracardiac major vessel lesions in complex CoA. Among them, transthoracic echocardiography (TTE) is the current first-line auxiliary diagnosis method. However, due to the low spatial resolution and poor imaging window of TTE, as well as the high requirements for cardiac sonographers, TTE is limited in evaluating extracardiac malformation^[19]^. Zhao’s study on dual-source computed tomography (DSCT) showed that DSCT could provide more reliable morphological features of large vessels and preoperative assessment of concomitant cardiac anomalies in children with aortic disease than TTE^[20]^. The 256-row Revolution™ CT is a noninvasive imaging technology that has emerged in recent years and has the characteristics of high spatial resolution, wide field of view, and image postprocessing technology. Our study showed that Revolution™ CT was accurate and reliable for the measurement and diagnosis of aortic diseases in infants, which could help surgeons intuitively and clearly understand the overall situation of aortic diseases and the spatial localization of surrounding vessels. In addition, the voltage used in the CT examination in this study was 70-80 kV, and the radiation exposure dose of the infants in this study was between 0.25 and 0.4 mSv, which is a low radiation exposure dose and had little impact on the infants. In addition, the results of this study also showed that Revolution™ CT could efficiently complete the preoperative examination and evaluation of CoA in infants. This was mainly due to the characteristics of Revolution™ CT’s wide-body detector and ultrahigh speed of 0.28 s, which could perfectly relieve constraints such as the baby’s rapid heart rate and the inability to cooperate with breath holding so that high-quality preoperative aortic images could be obtained without breath holding. Because Revolution™ CT could more clearly and accurately reflect the extracardiac structural abnormalities and the surrounding branch vessels, we would be more inclined to use the results of Revolution™ CT for preoperative evaluation in extracardiac structural abnormalities. We preferred a combination of the two methods for preoperative evaluation in infants with complex CoA.

### Limitations

This study has several limitations. First, this study was designed as a retrospective study. Due to the low incidence of the disease, we did not calculate the sample size. It was impossible to comprehensively collect some data that might greatly impact the outcome. For example, this study did not fully collect preoperative feeding conditions and could not assess the association between feeding conditions and outcomes. Second, as a single-center study, the results obtained might not necessarily apply to other centers. The small sample size also limits the accuracy of our findings. Finally, due to the short duration of our postoperative follow-up, the predictive accuracy of our results for the long-term postoperative prognosis of CoA in infants remains to be further investigated. More extensive multicenter studies are needed to determine the prognostic factors associated with complex CoA in infants.

## CONCLUSION

Revolution™ CT can provide a comprehensive and accurate preoperative evaluation of aortic diseases in infants with complex CoA. And the degree of CoA is an independent risk factor for prolonged postoperative CICU stay in infants with complex CoA.
